# OX40–OX40L Structural Insights Applied to Rocatinlimab in Atopic Dermatitis With Alopecia Areata Overlap: Mechanistic Hypotheses and Hair Regrowth

**DOI:** 10.1111/jocd.71227

**Published:** 2026-10-11

**Authors:** Waqar Iqbal Khan, Zerwah Zernab Malik, Zia Ullah, Zain Ul Abedeen, Kashmala Ali Yousafzai

**Affiliations:** ^1^ Department of Dermatology Saidu Medical College Swat Pakistan; ^2^ Department of Dermatology Shaikh Khalifa Bin Zayed Al‐Nahyan Medical and Dental College Lahore Pakistan; ^3^ Department of Dermatology Abbottabad International Medical College Abbottabad Pakistan

Dear Editor,

OX40 is a co‐stimulatory receptor which is responsible for activation and survival of T cells through OX40L, and therefore helps in maintaining Th1/Th2 mediated chronic inflammation. In atopic dermatitis (AD), raised OX40L expression exaggerates Th2 cytokine activity and disease activity, suggesting that blockade of OX40 could be useful in treatment [1]. In line with this concept, rocatinlimab, an anti‐OX40 monoclonal antibody, was developed as a T‐cell rebalancing therapy, where it selectively suppresses pathogenic T‐cell activity. It reduced disease severity, pruritus, skin involvement, sleep disruption, and quality‐of‐life impairment in moderate‐to‐severe AD, and clinical improvement was also observed after treatment discontinuation in the Phase 2b trial [2]. In March 2026, however, all ongoing rocatinlimab trials were discontinued after a planned safety review identified malignancies with possible viral or immune‐related links, including one new confirmed and one suspected case of Kaposi sarcoma in addition to a previously confirmed case. Although the overall number of malignancies remained below expected background rates, a biologically plausible safety concern could not be excluded. Rocatinlimab is therefore considered here as a mechanistic and translational lesson rather than a direct clinical recommendation [3, 4, 5].

Recent structural and epitope‐mapping findings in the *Journal of Investigative Dermatology* give more detail to this pathway. Molecular modeling and simulation work suggested that rocatinlimab and some other OX40/OX40L agents inhibit the interaction by physically blocking the OX40‐OX40L interface for ligand binding [6]. These findings help explain the molecular basis of receptor blockade, but they do not establish that this particular epitope provides greater clinical efficacy, deeper memory T‐cell depletion, or longer treatment durability. These potential effects are more appropriately considered consequences of OX40 pathway modulation and require direct clinical and mechanistic confirmation. These structural insights, together with clinical data from AD, provide a basis for considering whether OX40‐directed therapy may also be relevant to AD–AA overlap, although evidence in AA remains preliminary.

Alopecia areata (AA) and AD are immune‐mediated disorders with abnormal T‐cell responses. AA is mainly linked to CD8^+^NKG2D^+^ T cells and interferon‐gamma driven signaling, whereas AD is linked more to Th2‐mediated inflammation [7, 8]. Despite these differences, the OX40–OX40L pathway may provide a point of convergence at the level of T‐cell activation and persistence. In AD, OX40 signaling supports the activation, expansion, and survival of pathogenic T cells within a predominantly Th2‐skewed inflammatory environment [1, 7]. In AA, IFN‐γ/IL‐15 signaling and CD8^+^NKG2D^+^ cytotoxic T cells remain the better‐established drivers, although recent profiling has also demonstrated OX40‐axis activation across different atopic backgrounds [8, 9]. OX40 blockade could therefore influence the persistence of activated T‐cell populations in AA, but this remains a mechanistic hypothesis rather than an established therapeutic effect.

This overlap is important because AA is also a disease of hair‐follicle immune privilege collapse. In AA, cytotoxic T cells and IFN‐gamma dominant signaling contribute to immune attack around the follicle, while IL‐2/IL‐15 mediated common gamma‐chain signaling maintains inflammatory loops. JAK/IFN pathway blockade has shown that reducing pathogenic T‐cell activity can allow hair regrowth, supporting the idea that immune modulation may restore at least part of follicular immune privilege (Figure 1) [8].

**FIGURE 1:**
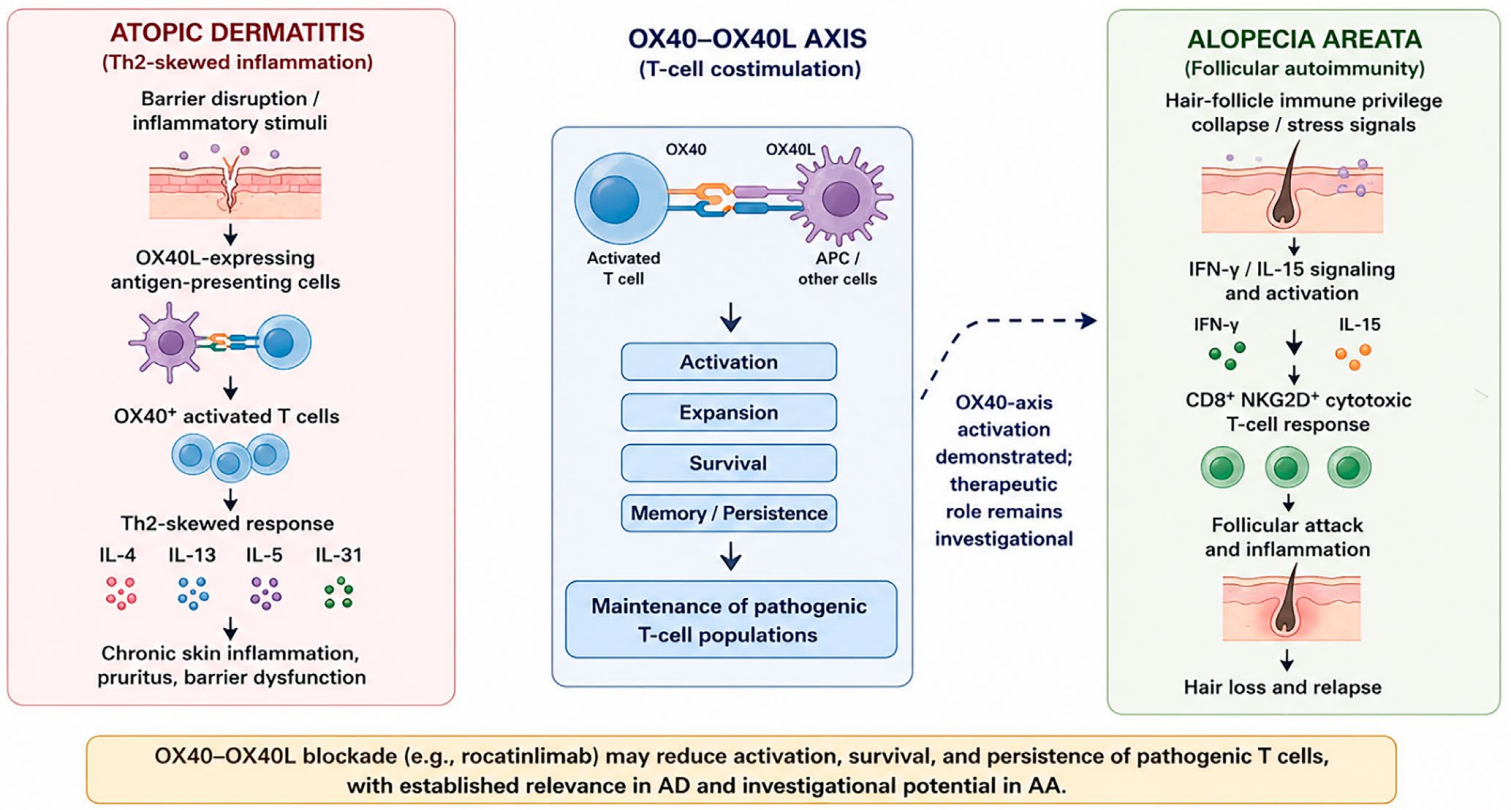
Proposed intersection of OX40–OX40L signaling in atopic dermatitis and alopecia areata. Atopic dermatitis is characterized predominantly by Type 2–skewed inflammation, in which OX40–OX40L co‐stimulation contributes to T‐cell activation, expansion, survival, and persistence. Alopecia areata is primarily associated with collapse of hair‐follicle immune privilege and IFN‐γ/IL‐15–dependent CD8^+^NKG2D^+^ cytotoxic T‐cell activity. OX40‐axis activation has also been identified in AA, providing a possible upstream point of convergence between the disorders. Its therapeutic relevance in AA, however, remains investigational [1, 7, 8, 9]. AA, alopecia areata; AD, atopic dermatitis; APC, antigen‐presenting cell; IFN, interferon; IL, interleukin; OX40L, OX40 ligand.

JAK inhibitors have established efficacy in AA by suppressing signaling through pathways such as IFN‐γ and IL‐15, but continued treatment is often needed to maintain hair regrowth [10, 11]. OX40 blockade differs by acting at the level of T‐cell co‐stimulation and may reduce the persistence of activated effector and memory T cells. This raises the possibility of more durable immune modulation, as suggested by off‐treatment responses with rocatinlimab in AD [2], but such an advantage has not been demonstrated in AA and no comparative trials with JAK inhibitors are available. Initial observations of hair regrowth with OX40‐directed therapy are encouraging, but they should be read cautiously. At present, this evidence is preliminary and includes anti‐OX40 agents other than rocatinlimab itself [12]. Dedicated AA trials and AD–AA overlap cohorts are needed before any conclusion about efficacy in hair loss can be made.

Rocatinlimab has shown an off‐treatment clinical effect in AD: in the Phase 2b trial, improvement was largely maintained among responders during the 20‐week period after treatment ended [2]. This suggests longer‐lasting immune modulation but does not prove treatment‐free remission or memory/T‐cell depletion. Comparable evidence is not yet available in AA, where anti‐OX40 clinical data remain preliminary [12]. Blocking OX40 signaling could therefore create longer immune regulation after active treatment, but the safety experience with rocatinlimab also shows that future OX40 programs would require predefined malignancy surveillance and long‐term safety follow‐up. This concern has biological plausibility because inherited OX40 deficiency has been associated with impaired CD4^+^ T‐cell memory and susceptibility to HHV‐8‐associated Kaposi sarcoma [13]. However, complete genetic OX40 deficiency is not equivalent to pharmacologic OX40 blockade, and whether anti‐OX40 therapy meaningfully compromises HHV‐8 immune surveillance remains uncertain. Future studies should not only report eczema outcomes, but also include hair‐specific and immune‐biomarker outcomes in patients with overlap disease.

Future precision immunotherapy studies should include combined dermatologic endpoints. EASI and pruritus outcomes should be assessed together with SALT score, patient‐reported hair outcomes, lesional and non‐lesional immune profiling, and biomarkers of memory T‐cell depletion or persistence. It would also be useful to examine whether OX40/OX40L blockade can restore follicular immune privilege or simply reduce inflammatory activity around follicles. AD–AA overlap may be a useful clinical model for this question because it brings barrier inflammation, autoimmunity, tissue‐resident memory, and follicular immune privilege in the same patient.

OX40 blockade remains a promising upstream approach for modulation of pathogenic T‐cell responses in AD and possibly AA, but it should be presented cautiously after rocatinlimab discontinuation. The rocatinlimab experience instead suggests a possible relationship between OX40 pathway modulation, pathogenic T‐cell persistence, and treatment durability, while the clinical significance of its specific structural epitope remains uncertain. Future trials of OX40/OX40L agents should therefore assess both skin and hair outcomes, while maintaining strong safety surveillance and translational biomarker follow‐up.

## Author Contributions

W.I.K.: conceptualization, writing – original draft, writing – reviewing and editing, project administration, validation. Z.Z.M.: conceptualization, writing – original draft, writing – reviewing and editing, validation. Z.U.: conceptualization, writing – original draft, writing – reviewing and editing, supervision, validation. Z.U.A.: writing – original draft, writing – reviewing and editing, resources, data curation, funding acquisition. K.A.Y.: writing – reviewing and editing, validation, data curation, visualization.

## Funding

The authors have nothing to report.

## Disclosure

*Provenance and Peer Review*: This study was not commissioned.

*Transparency Statement*: We affirm that this manuscript is an honest, accurate, and transparent account of the data being reported; that no important aspects of the perspective have been omitted.

## Ethics Statement

This study used publicly available data; no ethical approval was required.

## Consent

It is an Editorial, rendering publication consent irrelevant.

## Conflicts of Interest

The authors declare no conflicts of interest.

## Data Availability

Data sharing not applicable to this article as no datasets were generated or analyzed during the current study.
